# Assessment of Fractional-Order Arterial Windkessel as a Model of Aortic Input Impedance

**DOI:** 10.1109/OJEMB.2020.2988179

**Published:** 2020-04-22

**Authors:** Mohamed A. Bahloul, Taous-Meriem Laleg-Kirati

**Affiliations:** Computer, Electrical and Mathematical Sciences and Engineering DivisionKing Abdullah University of Science and Technology 127355 Thuwal Makkah 23955-6900 Saudi Arabia

**Keywords:** Arterial windkessel, vascular impedance, fractional calculus, fractional-order capacitor

## Abstract

*Goal:* Fractional-order Windkessel model is proposed to describe the aortic input impedance. Compared with the conventional arterial Windkessel, the main advantage of the proposed model is the consideration of the viscoelastic nature of the arterial wall using the fractional-order capacitor (FOC). *Methods:* The proposed model, along with the standard two-element Windkessel, three-element Windkessel, and the viscoelastic Windkessel models, are assessed and compared using in-silico data. *Results:* The results show that the fractional-order model fits better the moduli of the aortic input impedance and fairly approximates the phase angle. In addition, by its very nature, the pseudo-capacitance of FOC makes the proposed model's dynamic compliance complex and frequency-dependent. *Conclusions:* The analysis of the proposed fractional-order model indicates that fractional-order impedance yields a powerful tool for a flexible characterization of the arterial hemodynamics.

## I. Introduction

Over the last century, modeling of aortic input impedance has attracted significant interest, and several lumped parametric models have been proposed in this regard. Indeed, models of the aortic input impedance can serve to many purposes for clinical routine. For instance, they can be used as a tool for the estimation of physiological parameters, which are not easy to evaluate in an invasive way, like the total arterial compliance or the local arterial stiffness [1]. As a matter of principle, practical arterial models have to be simple and consist of interpretable characteristic parameters which are uniquely identifiable. In the open literature review, several reduced-order models have been proposed [2]. The well-known Windkessel ($\mathrm{WK}$) lumped-parametric model, with its different versions, was considered, for a long time, as an acceptable approximation of the aortic input impedance which can fit real data at low and high frequencies [3]. Besides, this paradigm involves physiologically interpretable elements. However, WK models present some limitations, such as the failure to represent all the arterial mechanical properties of interest accurately, such as the arterial stiffness [4]. Basically, similar to any bio-tissue, the arterial tissue presents a viscoelastic behavior rather than a pure elastic one [5]–[8]. Accordingly, real arterial compliance is considered to be complex and frequency-dependent. Nevertheless, most of the proposed $\mathrm{WK}$ models regard the arterial network as a pure elastic reservoir and represent the total arterial compliance using an ideal capacitor whose capacitance is constant over the whole cardiac cycle. Some research attempts have tried to overcome this limitation by connecting a small resistance in series with an ideal capacitor, resulting in a complex and frequency-dependent capacitance (compliance) [9]. The later configuration is based on the mechanical Voigt viscoelastic model that consists of a spring connected in parallel to a dashpot, accounting for the static compliance and viscous losses, respectively [10]. Even though, many studies have argued that the Voigt model is a poor representation since it does not account for the stress-relaxation experiment, this representation is commonly adopted [11]. This is related to the fact that, even if higher-order viscoelastic models would provide a more realistic representation, the real aortic input impedance cannot depict sufficient information to identify all the parameters of these complex models. Conventional integer-order lumped parameter models used to simulate the viscoelastic properties of such bio-tissues are, usually, not sufficient, as they do not account for the power-law demonstrated experimentally in viscoelastic materials [12], see Suppl. Materials (S.I) and Fig. S1.

In the last decade, fractional-order derivative (FD), defined as the generalization of the standard integer derivative to a non-integer order, has gained paramount popularity in modeling and characterizing biological tissues [13], [14]. Because of its non-locality and memory properties, FD has been considered as a powerful tool for modeling complex physical phenomena that exhibit power-law response or involve memory effects [15]. Recently, the power-law behavior has been demonstrated in the viscoelasticity characterization of an elastic aorta. The *in-vivo* and *in-vitro* data analysis showed that FD-based models are more convenient to accurately describe the arterial viscoelastic dynamic response [16]–[19]. In addition, recent investigations have revealed the key advantages of using FD tools to accurately describe the viscoelasticity properties of collagenous tissues in the arterial bed; to analyze the arterial blood flow [20], [21]; to characterize the red blood cell (RBC) membrane mechanics [22] and to represent the heart valve cusp [23]. Bearing the above thoughts in mind, the application of FD for probing mechanical properties of the arterial system seems to be in accordance with their physical nature. Additionally, incorporating the fractional-order element for describing the aortic input impedance appears to be a very potent appliance, to interpret physiological phenomena. In this article, we derive a fractional-order two-element arterial Windkessel model (FWK2) for describing the aortic input impedance. The proposed model uses the same model structure as the well-known two-element Windkessel, but by replacing the ideal capacitor with a fractional-order capacitor, see Suppl. Materials (S.II) and Fig. S1 (D). FWK2 offers a paradigm shift in the development and the characterization of the arterial Windkessel, allowing greater flexibility in characterizing the arterial dynamics. A detailed comparison between the proposed model and the conventional WK models in terms of the ability to describe the aortic input impedance spectrum is demonstrated. Furthermore, the correlation between the proposed model's dynamic compliance and the standard Windkessel models’ compliance has been discussed. The mathematical derivations and the detailed results obtained using in-silico data of 3325 virtual subjects. are given in the following sections.

## II. Materials and Methods

### A. In-Silico Hemodynamic Data

**Fig. 1.**
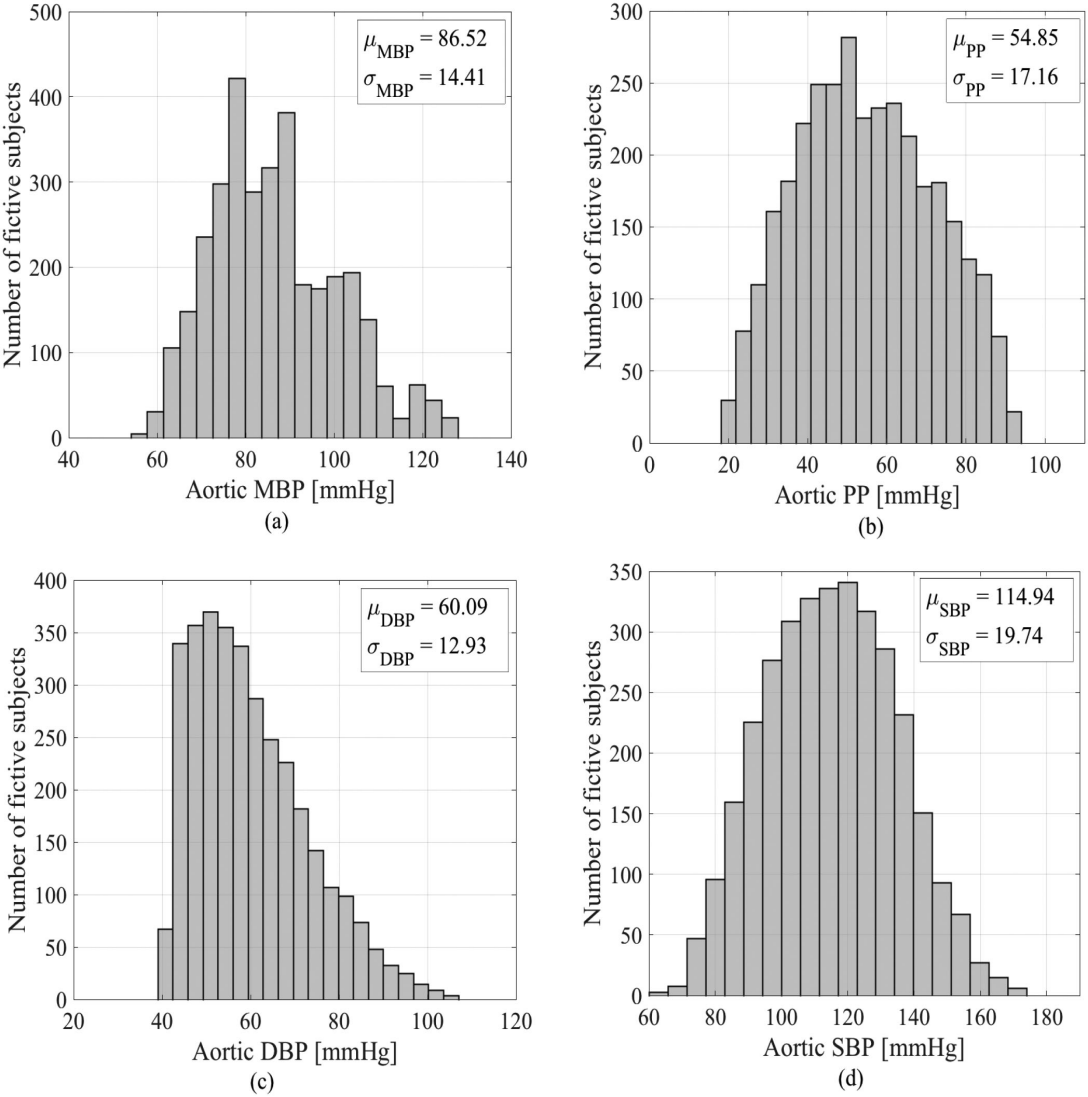
Distribution and summary statistic (mean and standard deviation values) of: (a) mean blood pressure (MBP) in (mmHg), (b) pulse pressure (PP) in (mmHg), (c) diastolic pressure (DP) in (mmHg) and systolic pressure (SP) in (mmHg) at the level of of ascending aorta for the in-silico database.

The proposed model has been validated using the hemodynamic in-silico data set, generated by Willemet *et al.*, [24], with a validated one-dimensional numerical model of the arterial network (Nektar1D, [25]). This database consists of hemodynamic signals (e.g., pressure, flow, and distension waveforms) at all arterial locations. It presents arterial hemodynamic of 3325 virtual healthy adult subjects whose cardiac and arterial parameters vary within physiological ranges. This in-silico data set can mimic the main hemodynamic properties sensed *in-vivo*.^1^^1^http://haemod.uk/virtual-database. For this study, we use in-silico blood pressure and flow waveforms generated at the ascending aorta site. Fig. 1 shows a summary statistic of the features of the blood pressure signal for all virtual subjects. Based on these plots, it is clear that this database exhibits physiological values with well-balanced distributions. The cardiac outputs range between $\mathrm{3.5}$ and $\mathrm{7.2 \ l/min}$, depending on the values of the heart rate $\mathrm{(53, \ 63, \ and \ 72 \ beats/min)}$ and stroke volume $\mathrm{(66, \ 83,\ and \ 100 \ ml)}$ prescribed.

### B. Proposed Fractional-Order Two-Element Windkessel

Based on the conservation mass, the arterial blood flow, $Q_{a}$(t), pumped from the left ventricle to the arterial vascular bed can be expressed as:

\begin{equation*}
Q_{a}(t)=Q_{stored}(t)+Q_{out}(t), \tag{1}
\end{equation*}

where $Q_{stored}(t)$ is the blood stored in the arterial tree, and $Q_{out}(t)$ corresponds to the flow out of the arterial system. $Q_{out}(t)$ is supposed to be proportional to the aortic blood pressure $P_a(t)$, that is:

\begin{equation*}
Q_{out}(t)=\frac{1}{R_{p}}P_a(t). \tag{2}
\end{equation*}

Regarding $Q_{stored}(t)$, typically using the standard definition, it can be determined as the rate of flow by taking the first derivative of the volume $V(t)$ equation for the time, whereas, in consideration of the fractional properties of both RBC and the collagenous tissues forming the arterial bed, we allow the differentiation order of the blood volume for time to be real, $\alpha \in [0\ 1]$, and hence applying the fractional-order derivative to this differential equation.

\begin{align*}
Q_{stored}(t)&=D_t^{\alpha } V(t)= \frac{{d^{\alpha }V(t)} }{{dt^{\alpha }}} \tag{3}\\
Q_{stored}(t)&= \underbrace{\frac{d^{\alpha }V(t)}{d^{\alpha }P(t)}}_{C_{\alpha }} \frac{{d^{\alpha }P(t)} }{{dt^{\alpha }}}=C_\alpha \frac{{d^{\alpha }P(t)} }{{dt^{\alpha }}} \tag{4}
\end{align*}

where $ C_\alpha \!\!=\!\! \frac{d^{\alpha }V(t)}{d^{\alpha }P(t)}$ is a fractional order proportionality constant that is expressed in the unit of $\mathrm{[l/mmHg. sec^{1-\alpha }]}$. Substituting (2) and (4) into (1) yield:

\begin{equation*}
Q_{a}(t)=C_\alpha \frac{{d^{\alpha }P_a(t)} }{{dt^{\alpha }}}+\frac{1}{R_{p}}P_a(t). \tag{5}
\end{equation*}

Assuming null initial condition, the $Laplace$ transform of (5) is given as:

\begin{equation*}
Q_{in}(s)= C_\alpha s^\alpha P+\frac{1}{R_{p}}P. \tag{6}
\end{equation*}

Hence the fractional-order aortic input impedance, which is defined as input blood pressure-to-flow rate ratio in the frequency domain, can be expressed as:

\begin{equation*}
Z_{in}^{FWK2}=\frac{R_p}{1+R_p C_\alpha s^\alpha }. \tag{7}
\end{equation*}

### C. Numerical Implementation and Models’ Calibration

In this part, we present the general framework for identifying models of aortic input impedance based on what was introduced by Graupe [26] for the frequency response technique. An acceptable lumped-parametric model of the vascular impedance must satisfy two main conditions [27], which are:

• **Identifiability:** The developed model should be structurally identifiable. In other words, the mathematical formulation of the model should guarantee the uniqueness of the parameters estimates. Usually, to satisfy this condition, we look for data-driven or black box models that provide an overall approximation of the observed central blood flow-pressure relationship (aortic input impedance), in the frequency domain. It is worth to mention that, in this step, no specific information about how the physiological system operates and gives rise to the input/output behavior, is needed. Usually, the generalized form of arterial Windkessel models is formulated as a gain ($G$) multiplied by a ratio between zeros and poles, as follow:
  \begin{equation*}
Z_{in}=G\frac{1+\sum _1^N a_is^i}{1+\sum _1^M b_is^i}, \tag{8}
\end{equation*}
  where, ($N \in \mathbb {N}$) and ($M \in \mathbb {N}$) are the numbers of zeros and poles, respectively. For example, for WK models, usually, ($N,M$) are less or equal to 2. The coefficients $a_i$ and $b_i$ ($i=1..N$) are purely phenomenological.
• **Interpretability:** To satisfy this condition, usually, we search for an appropriate analog structure that is suitable with the identified frequency response, and consists of parameters that are representative of physiological attributes, serving in interpreting the physical processes, and matching with the experimental observations.

**Fig. 2.**
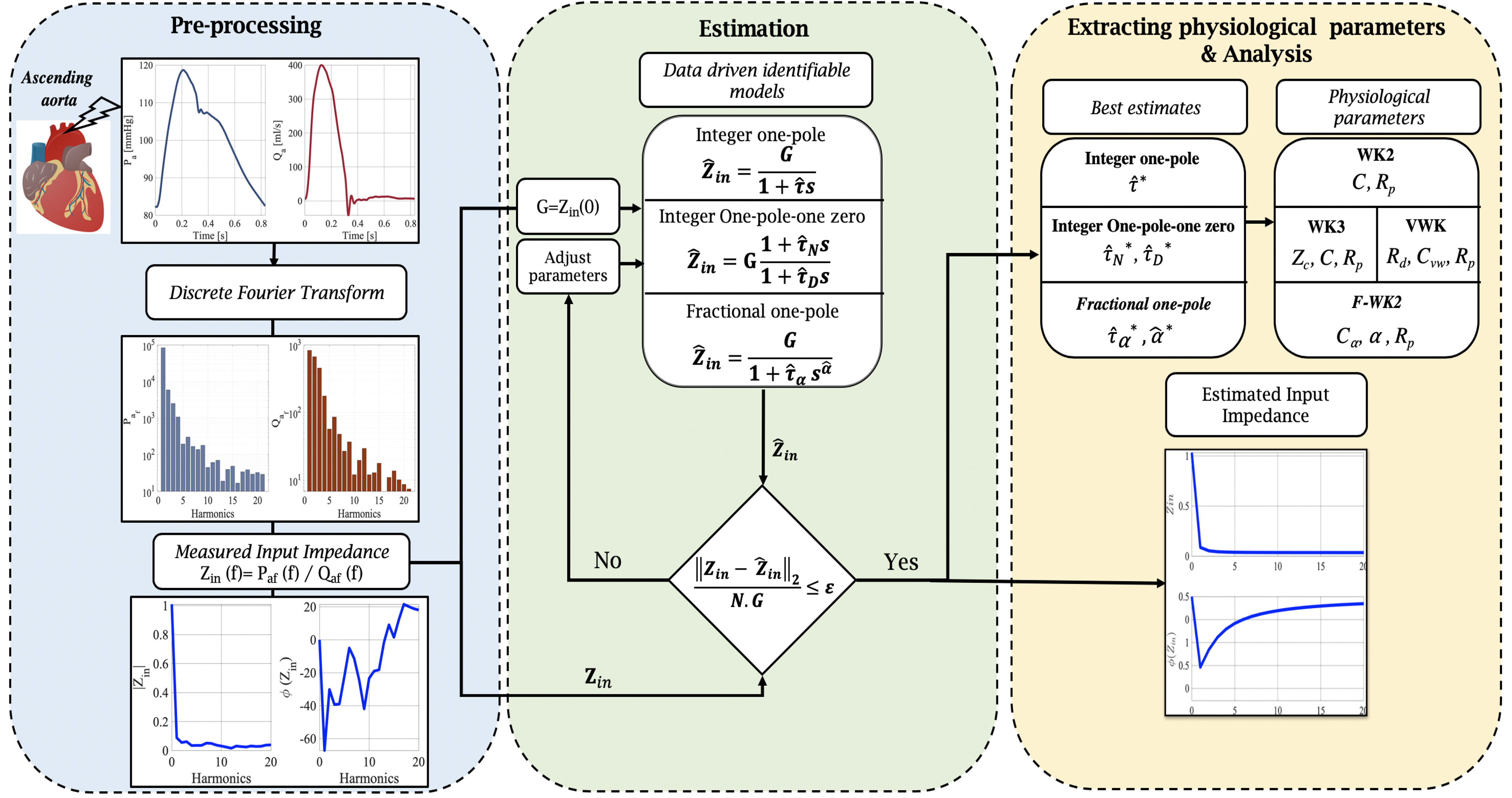
A flowchart outlining the framework of the proposed FWK2 model calibration and comparison with the standard Windkessel models (WK2, WK3, and VWK).

Following the modeling guidelines introduced above, the general procedure used to validate the proposed model and to compare its performance to standard Windkessel models (WK2, WK3 and, VWK) in estimating the aortic input impedance spectrum, is depicted in Fig. 2

and given in the following steps:

#### 1) Pre-Processing

In this step, the in-silico pressure, $P_a (t)$, and flow, $Q_a(t)$, at the level of the ascending aorta are collected. Then, these two hemodynamic signals are decomposed using *Fast Fourier Transform* (FFT), which translates them into a sum of their mean value and a series of sinusoidal components at a specific frequency, according to the following expressions:

\begin{align*}
P_a(t)&=P_{a_0}+\sum _{n=1}^{N}P_{a_n}\cos (n\omega _0 t+\beta _n), \tag{9}\\
Q_a(t)&=Q_{a_0}+\sum _{n=1}^{N}Q_{a_n}\cos (m\omega _0 t+\Theta _n). \tag{10}
\end{align*}

In the above expressions, $P_{a_0}$ and $Q_{a_0}$ correspond to the mean value of the aortic pressure and flow respectively; $n$ indicates the $n^{th}$ harmonic of the flow and pressure in the Fourier series; $N$ is the total number of harmonics; $P_{a_n}$ and $Q_{a_n}$ are the amplitudes of the $n^{th}$ sinusoidal component of the aortic pressure and flow, respectively, and $\beta _n$ and $\Theta _n$ are the related phase angles; $\omega _0$ is the fundamental frequency of the cardiac pulsation in the unit of [rad/sec] ($\omega _0=2\pi /T$ where $T$ is the heart period). Each term $P_{a}(jnw)$ and $Q_{a}(jnw)$ obtained by FFT can be expressed as function of its modulus, $P_{a_n}$ and $Q_{a_n}$ and its phase angle, $\beta _{n}$ and $\Theta _{n}$ as follows:

\begin{align*}
P_{a}(jnw_0)&=P_{an}[e^{j(n\omega _0 t+\beta _n)}], \tag{11}\\
Q_{a}(jnw_0)&=Q_{an}[e^{j(n\omega _0 t+\Theta _n)}]. \tag{12}
\end{align*}

Subsequently, harmonic by harmonic, the in-silico aortic input impedance is evaluated as the ratio of $P_{a}(jnw_0)$ to $Q_{a}(jnw_0)$:

\begin{equation*}
Z_{in}(jnw)=\frac{P_{an}}{Q_{an}}\frac{[e^{j(n\omega _0 t+\beta _n)}]}{[e^{j(n\omega _0 t+\Theta _n)}]}= Z_n e^{j\Phi _n} \tag{13}
\end{equation*}

where $\Phi _n$ is the phase angle of $Z_{in}$ as the difference between $\beta _n$ and $\Theta _n$, and $Z_n$ is its modulus as the ratio between $P_{an}$ and $Q_{an}$.

#### 2) Estimation Phase

The subsequent step, after preparing the in-silico aortic input impedance, is to fit the pre-processed data to an identifiable model that satisfies the first condition. To do so, for the proposed FWK2 model, we fit the aortic input impedance in-silico data to a fractional-order *one-pole* transfer function that is:

\begin{equation*}
H_\alpha (s)=G\frac{1}{1+\tau _\alpha s^\alpha }, \tag{14}
\end{equation*}

where $G$, $\alpha$, and $\tau _\alpha$ are the observational coefficients to be estimated. In [28], the structural global identifiability of $H_\alpha$, has been proved, which ensures the uniqueness of the coefficients’ estimates. In addition, $G$, $\tau _\alpha$, and $\alpha$ depend on the characteristic parameters of $Z_{in}^{FWK2}$ as:

\begin{equation*}
\left\lbrace \begin{array}{l}
G=R_p\\
\tau _\alpha =C_\alpha R_p \end{array}\right. \tag{15}
\end{equation*}

For WK2, WK3, and VWK arterial models, the identifiable transfer functions are given in Appendix B, Suppl. Materials.

In this study, for all the considered models, the gain $G$ was evaluated as:

\begin{equation*}
G=\frac{P_{a_0}}{Q_{a_0}}=Z_{in}(0). \tag{16}
\end{equation*}

The remaining model parameters, $\theta _{model}$ that is $\lbrace \tau _\alpha, \alpha \rbrace$ for FWK2, were estimated via solution of the inverse problem of the estimated aortic input impedance ($\hat{Z}_{in}$) and the in-silico one ($Z_{in}$). Initialized by ${\theta _0}$, the inverse algorithm iteratively predicts the set of parameters $\hat{\theta }$ which minimizes the normalized root mean square error (NRMSE) between the complex $Z_{in_{[i]}}$ and the model predicted $\hat{Z}_{in_{[i]}}(G,\theta)$ evaluated at the $i^{th}$ harmonic. The objective function NRMSE is defined in equation (17) shown at the bottom of this page.

\begin{align*}
\mathrm{NRMSE}=\frac{\sqrt{\sum _{i=0}^{N}\left\lbrace \left[Real(Z_{in_{[i]}})-Real(\hat{Z}_{in_{[i]}}(G,\theta))\right]^2+\left[Imag(Z_{[i]})-Imag(\hat{Z}_{in_{[i]}}(G,\theta))\right]^2 \right\rbrace }}{(N+1) \ \ G} \tag{17}
\end{align*}

In this expression, *Real* and *Imag* denote the real and imaginary parts, respectively, and $N$ is the number of harmonics. Because it has been proven that the frequency range of physiological interest is from 0 Hz to 20 times the heart rate, here we choose $N$ to be equal to 20. It is worth mentioning that each harmonic is a multiple of the heart rate based on Fourier method resolution. To numerically implement the objective function, NRMSE, a constrained method (*fmincon* function in MATLAB), was used. Using this function, we constrained the parameters to be positive to guarantee physical properties. Once a tolerance of error was reached, the convergence of the method is confirmed, the *fmincon* function exits, and yields an output of the optimal set of model parameters estimates ${\hat{\theta }^*}$. In addition to NRMSE and to assess the performance of FWK2 method as well as WK models, the deviation of the moduli from the in-silico aortic input impedance moduli was evaluated, using the following expression:

\begin{equation*}
\mathrm{D_{[i]}\ [\%]}=\left[ \frac{\left| \hat{Z}_{in_{[i]}}(G,\theta) \right|-\left|Z_{in_{[i]}} \right|}{\left| Z_{in_{[i]}}\right|}\right]_{i=1..N}\times 100 \%. \tag{18}
\end{equation*}

For ease of visualization of the various comparisons between the different models, for every subject, we assessed the mean of $D [\%]$ over the ($N+1$) harmonics, using the following equation:

\begin{equation*}
\mathrm{Deviation\ [\%]}=\frac{\sum _{i=0}^{N}D_{[i]} [\%]}{N+1}. \tag{19}
\end{equation*}

Furthermore, the effectiveness of each model is evaluated by calculating the relative error (RE %) between the real moduli and phase of $Z_{in}$, and their estimates as follows:

\begin{equation*}
\left\lbrace \begin{matrix}
\mathrm{\underset{moduli}{Re} (\%)}=\frac{\left\Vert |Z_{in}|-|\hat{Z}_{in}|\right\Vert _2}{\left\Vert |Z_{in}| \right\Vert _2} \times 100 \\
\mathrm{\underset{phase}{Re}(\%)}=\frac{\left\Vert \angle Z_{in}-\hat{\angle Z_{in}}\right\Vert _2}{\left\Vert \angle Z_{in} \right\Vert _2} \times 100 \end{matrix}\right. \tag{20}
\end{equation*}

The approximated proposed fractional-order model-based input impedance moduli and phase angle at a specific radial frequency $ {\omega }$ can be evaluated based on these expressions:

\begin{align*}
\left| {Z}_{in}^{FWK2}\right|\!&=\!\!\frac{G}{\sqrt{[1\!+\!\omega ^{\alpha }\tau _\alpha \cos (\alpha \frac{\pi }{2})]^{2}\!+\![\omega ^{\alpha }\tau _\alpha \sin (\alpha \frac{\pi }{2})]^{2}}}, \tag{21}\\
\angle {Z}_{in}^{FWK2}&=-\tan ^{-1}\left(\frac{\omega ^{\alpha }\tau _\alpha \sin (\alpha \frac{\pi }{2})}{1+\omega ^{\alpha }\tau _\alpha \cos (\alpha \frac{\pi }{2})} \right). \tag{22}
\end{align*}

#### 3) Extracting Physiological Parameters & Analysis

For the sake of physiological analysis of model parameters, and to take advantage of the identified frequency response, this step focuses on the reconstruction of a compatible analog structure which helps in parsing and interpreting the physiological process. Indeed, this phase satisfies the interpretability condition explained above. Accordingly, after fitting the experimental data, the assumption of the model structure becomes essential if we desire to interpret the features physiologically, and characterize the reference aortic input impedance based system. Subsequently, based on the optimal estimates of $\hat{\theta }^*$, we try to extract the physical parameters of the corresponding structure. With respect to the proposed FWK2 model, Fig S1. (D) shows the appropriate structure to the identifiable function $H_\alpha$. This structure comprises a resistor, $R_p$, connected in parallel to a FOC, $C_\alpha$, representing the total peripheral resistance and the complex and frequency-dependent arterial compliance, respectively. Based on the transfer function $Z_{in}^{FWK2}$, the characteristic physical parameters, $R_p$, $C_\alpha$, and $\alpha$, are evaluated from the optimal estimation of the observational coefficients of $H_\alpha$ as follow:

\begin{equation*}
\left\lbrace \begin{matrix}
R_p=\hat{G}^*\\
C_\alpha = \frac{\hat{\tau }_\alpha ^*}{\hat{G}^*}\\
\alpha =\hat{\alpha }^* \end{matrix}\right. \tag{23}
\end{equation*}

## III. Results

### A. FWK2 Model Based Aortic Input Impedance Estimation

**Fig. 3.**
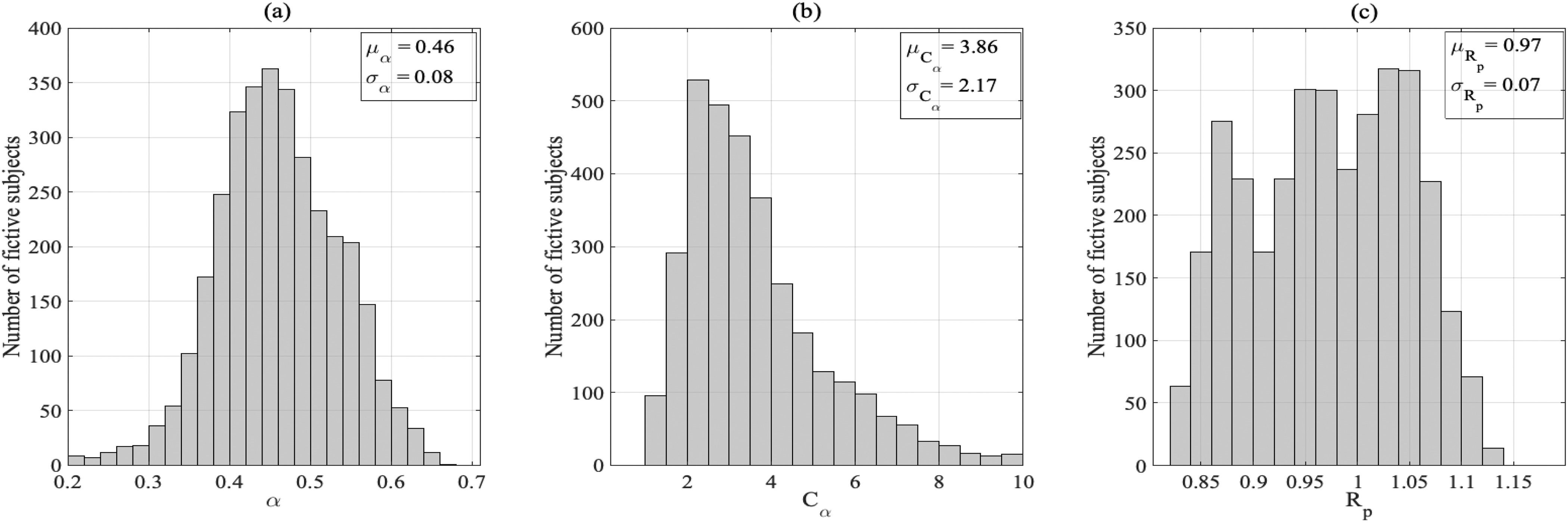
Distribution, mean value and standard deviation of the FWK2 parameter estimates: (a) the fractional differentiation order parameters $\alpha$, (b) the pseudo-compliance $\tau _\alpha$, and (c) the peripheral resistance ($R_p$).

Fig. 3(a), (b), and (c) show the distribution of the physical parameters estimates of the FWK2 model, after fitting the in-silico data of the aortic input impedance. By observing the distribution of the fractional differentiation order, $\alpha$, estimates, it is clear that this parameter is less than 1 for all the 3325 patients. Its mean value is approximately $0.46 \pm 0.008$. It is worth noting that in the estimation phase, for the parameter $\alpha$, we have only constrained the lower bound to be zero; however, for the upper bound, it was unconstrained. Accordingly, this result indicates that the arterial system exhibits a viscoelastic behavior, not a purely elastic one. Indeed, the fact that $\alpha \ne 1$ implies that the FoC element incorporates both resistance and capacitance behaviors, see Suppl. Materials (S.II). This result further supports the concept of fractional-order behavior by the arterial system. In the proposed model, the fractional-order element combines both the resistance and the capacitance properties, which display the viscoelastic behavior of the arterial vessel. The contributions from both properties are controlled by the fractional differentiation order $\alpha$, enabling a more flexible physiological description. As the fractional power approaches to 1 the capacitance part dominates and, hence the arterial system behaves like a pure elastic system.

### B. Comparison of Aortic-Input Impedance Estimation From WK2, WK3, VWK and FWK2 Models

The mean values of the goodness of fit criterion ($\mathrm{NRMSE},\mathrm{\underset{moduli}{Re}(\%)},\mathrm{\underset{phase}{Re}(\%)}$, $\mathrm{Deviation (\%)}$) of each group, after applying all the models, are reported in Table 1. It is clear, that the proposed model, FWK2, fits better the in-silico aortic input impedance than the classical WK2 model, overall. In fact, the averaged $\mathrm{NRMSE}$ over the three groups was reduced from ($1.06\cdot 10^{-2}\pm 0.27\cdot 10^{-2}$) using WK2 to ($0.6\cdot 10^{-2}\pm 0.11\cdot 10^{-2}$) using FWK2. Likewise, the mean values of the relative errors of both moduli and phase as well as the deviation of the magnitude of the in-silico aortic input impedance decreases significantly. In comparison to WK3 and VWK, the proposed FWK2 performs slightly better in terms of deviation of the aortic input impedance magnitude. In fact, the averaged value of the deviation over the three groups is about $16.04 \pm 5.34$ for FWK2 and $18.57 \pm 4.96$ with respect to WK3 and VWK. Nevertheless, FWK2 still performs poorly when trying to predict the phase angle of the aortic input impedance. This is due to the fact that, the phase angle of the whole model structure converges asymptotically to ($-\alpha \frac{\pi }{2}$) when the frequency tend to go to infinity and since $\alpha$ is between 0 and 1, the phase angle of the estimated aortic input impedance will never exceed $0^\circ$. However, the phase angle of the real aortic input impedance shows a pattern that goes beyond $0^\circ$.

**Table I:** Mean Values of the Goodness of Fit Criterion ($\mathrm{NRMSE},\mathrm{\underset{moduli}{Re}(\%)},\mathrm{\underset{phase}{Re}(\%)}$, and $\mathrm{Deviation (\%)}$) of Each Heart Rate Based-Group

	**WK2**				**WK3 & VWK**				**FWK2**			
**HR**	NRMSE ($\times 10^2$)	$\mathrm{\underset{moduli}{Re} }$ [%]	$\mathrm{ \underset{phase}{Re}}$ [%]	Deviation [%]	NRMSE ($\times 10^2$)	$\mathrm{\underset{moduli}{Re}}$ [%]	$\mathrm{\underset{phase}{Re}}$ [%]	Deviation [%]	NRMSE ($\times 10^2$)	$\mathrm{\underset{moduli}{Re} }$ [%]	$\mathrm{\underset{phase}{Re}}$ [%]	Deviation [%]
**53**	1.10$\pm$0.32	13.67$\pm$3.42	273.03$\pm$50.80	61.08$\pm$5.90	0.37$\pm$0.12	4.67$\pm$1.47	46.94$\pm$14.40	17.87$\pm$3.82	0.6$\pm$0.12	4.92$\pm$1.21	96.75$\pm$12.79	15.36$\pm$4.47
**63**	1.09$\pm$0.27	12.94$\pm$3.00	247.58$\pm$48.39	58.55$\pm$5.36	0.35$\pm$0.11	4.48$\pm$1.34	43.33$\pm$13.70	19.00$\pm$5.86	0.6$\pm$0.11	4.97 $\pm$ 1.21	91.03$\pm$13.87	16.44$\pm$6.26
**72**	1.00$\pm$0.24	12.37$\pm$2.85	234.04$\pm$44.11	56.92$\pm$5.00	0.32$\pm$0.11	4.43$\pm$1.51	41.14$\pm$12.46	18.84$\pm$5.21	0.6$\pm$0.12	5.42$\pm$1.50	91.03$\pm$13.92	16.34$\pm$5.3

**Fig. 4.**
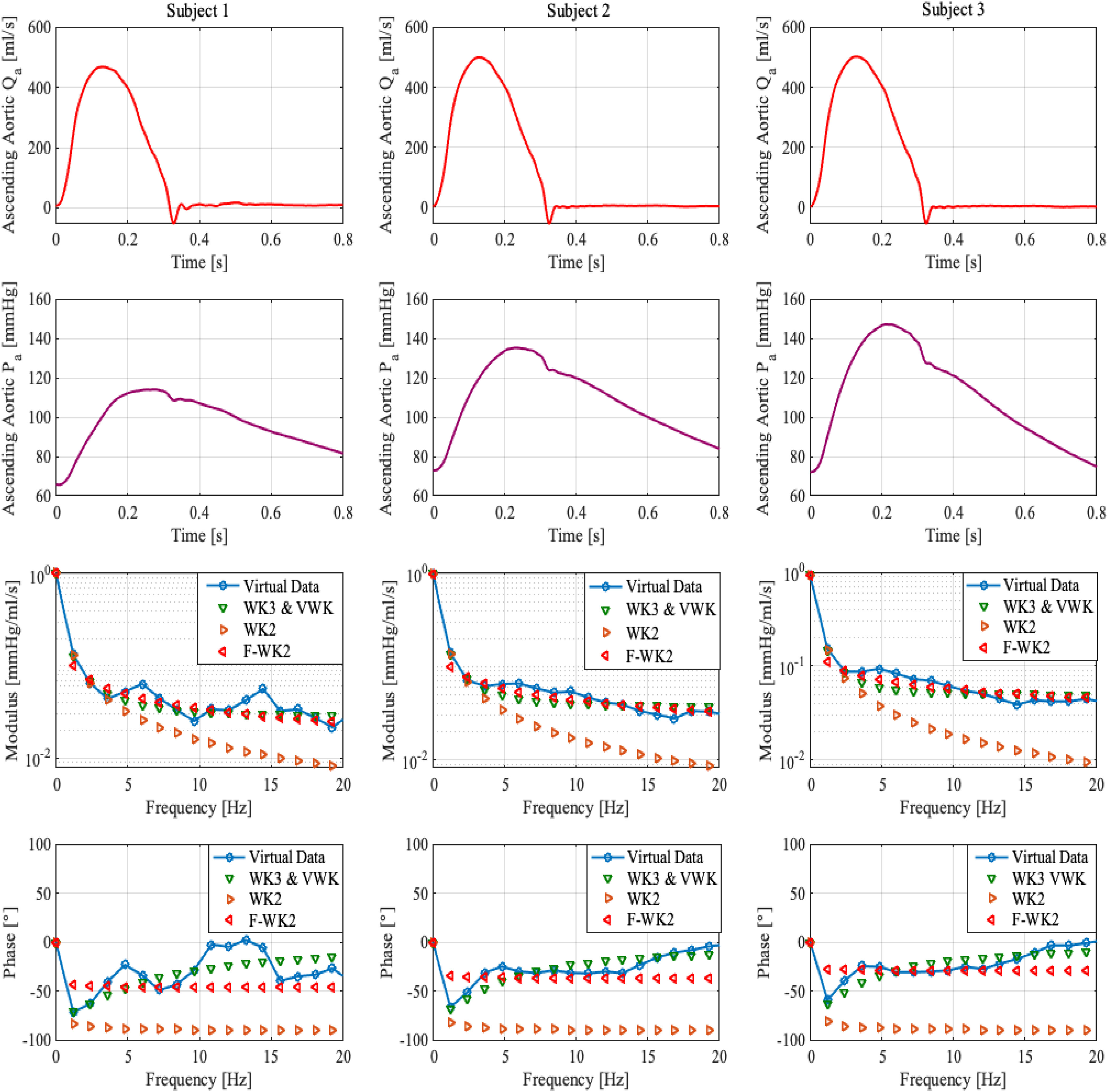
Examples of the validation of the in-silico impedance modulus and phases using the proposed model (FWK2) along with the standard Windkessel models (WK2, WK3, and VWK). The first and second rows represents, respectively, the blood flow ($Q_a$) and pressure ($P_a$) at the level of the ascending aorta for three different virtual subjects. The approximated impedance modulus and phase angles, along with the in-silico one, are depicted in the below figures.

Fig. 4 shows examples of the validation of aortic impedance modulus and phases for three different virtual subjects having various hemodynamic features. The diastolic, systolic, pulse pressure values, and the fractional-order estimates, $\mathrm{\alpha }$, for each subject, are reported in Table 2. These results are consistent with the conclusions aforementioned. Indeed, the table shows that $\mathrm{FWK2}$ model is significantly improving the prediction of the in-silico input impedance moduli comparing to the WK2 model and performing closely to $\mathrm{WK3}$ and $\mathrm{VWK}$. The variation of $\mathrm{\alpha }$ values from a physiological state to another shows that the differentiation order ${\alpha }$ is correlated to all the arterial parameters. For instance, as the diastolic pressure, systolic pressure, and pulse pressure values increase, the estimate of $\alpha$ decreases. Hence, on one hand, the fractional differentiation order may reflect physiological insights where a decrease in $\mathrm{\alpha }$ means an increase in the resistance part. On the other hand, one of the severe causes of hypertension (elevated blood pressure) is augmenting arterial stiffness [7]. In this regard, the new parameter $\alpha$ can be explored as a novel biomedical indicator that lumps the overall viscoelasticity properties of the human arterial tree. Accordingly, $\alpha$ might help to understand the arterial stiffness dependencies in a better way.

**Table II:** Blood Pressure Based-Features (Diastolic, Systolic, and Pulse Pressures Amplitude Values) and the Corresponding Fractional Differentiation Order $\alpha$ Estimate in Three Different Virtual Subjects

	$\mathrm{Diastolic \ Pressure }$ [mmHg]	$\mathrm{Systolic \ Pressure }$ [mmHg]	$\mathrm{Pulse \ Pressure}$ [mmHg]	$ {\alpha }$
**Subject 1**	65.63	114.23	48.60	0.52
**Subject 2**	72.79	135.45	62.46	0.42
**Subject 3**	72.16	147.17	75.24	0.33

## IV. Discussion

In the light of the above results, the need for a new model, namely FWK2, as an alternative to the ordinary arterial Windkessel models, in particular the VWK model, can be criticized for the following reasons:

• Previously, the WK3 arterial model has been proposed as an alternative to overcome the discrepancies of WK2 in terms of data-fit of the magnitude and phase angle of the aortic input impedance.
• Subsequently, for the sake of attributing relevant physical interpretation of the model's parameters, VWK model came as alternative to WK3 by taking into account the viscoelastic property of the arterial wall. The latter considered the systemic arterial system as a viscoelastic reservoir rather than a pure elastic one and adopted the Voigt cell structure to represent the dynamic compliance.
• The investigations of FWK2 might be worthless, since VWK has proven its reliability in terms of data-fit results and physical interpretability of the characteristic parameters.

The above criticisms can be addressed by the following observations:

• Although the VWK arterial model used to yield to an acceptable representation of the arterial system, the use of Voigt configuration leads to a poor modeling of the arterial compliance dynamics. This is because the Voigt model does not account for the stress-relaxation response. Even though in the interest of proposing a more realistic representation of viscoelasticity, higher-order models which combine several Voigt cells have been proposed. This solution appeared to not be practical. Indeed, the real aortic input impedance data does not portray enough information to identify the large number of parameters of these complex structures [11].
• Recent experimental studies have demonstrated that FD is a suitable tool for probing the viscoelasticity of collagenous tissues in the arterial bed [17]. In fact, fractional-order framework allows the reduction of the complexity order and further improve the modeling accuracy. In this regards, mechanical fractional-order viscoelasticity models ($\mathrm{FVM}$), such as the spring-pot element (fractional-order mechanical component) have been used to correctly match the frequency morphology in arteries [29]. $\mathrm{FVM}$ usually contains one or two spring-pots. This fractional element displays the fractional-order derivative relationship between the mechanical stress $ {(\sigma (t))}$ and strain $ {(\epsilon (t))}$ on the vessel as described in the following:
  \begin{equation*}
\sigma (t)=\eta D^\alpha _t \epsilon (t) \tag{24}
\end{equation*}
  where ${\alpha }$ is the fractional differentiation order coefficient which controls the level of viscoelasticity of the artery, and $ {\eta }$ is a constant of proportionality. As $\mathrm{\alpha }$ approaches to $\mathrm{1}$, the artery's behavior is similar to a pure viscous dashpot (resistance dominance), and when $\mathrm{\alpha }$ border on $\mathrm{0}$, the vessel wall motion is more similar to that of a pure elastic spring [13], [16].
• *In-vivo* investigations have shown that the fractional-order differentiation order, $\alpha$, is a key parameter as it is associated with smooth muscle (SM) activity that modulates the viscoelasticity in arteries. In fact, SM cells stretch collagenous fibres and hence, vascular activation modifies the local viscoelastic response of the arterial wall [17]. Accordingly, a clear power-law response was shown in the frequency response to estimate Young's modulus in the range 0-100Hz [18], [19].

Conclusively, a key missing item for the lumped-parametric arterial method is a realistic fractional-order concept which can represent physiological behaviors. In this regard, FWK2 would represent a paradigm shift in hemodynamic modeling.

### A. The Position of $\mathrm{FWK2}$ Model in Representing Dynamic Compliance

Arterial compliance describes the arterial capacity to store blood in the entire systemic arteries. Functionally, it is equal to the variation in blood volume ($dV$) divided by the variation in the systemic input pressure ($dP_{in}$). However, the relationship between the arterial blood volume and input pressure $(dV/dP_{in}$) is not only governed by the total arterial compliance but also incorporates the effect of pulse wave reflection. In fact, $dV/dP_{in}$ relationship is equivalent to the true arterial compliance $C_{true}$ only at low frequency (i.e., $w\leq heart \ rate$). Hence, the concept of dynamic arterial compliance-or, equivalently, apparent compliance ($C_{app}$) has been proposed by Quick $et \ al.$ [30] to show how to correctly estimate the true compliance, $C_{true}$, from the transfer function $C_{app}=dV/dP_{in}$ [31]. For $\mathrm{WK2}$ and $\mathrm{WK3}$, it is clear that this transfer function is thought to be a constant (scalar) and, is modeled by a constant capacitance of an ideal capacitor which yields to inaccurate evaluation of the true arterial compliance. Nevertheless, in $\mathrm{VWK}$ the small resistor added in series to the ideal capacitor yields to a reconstruction of an equivalent frequency-dependent compliance that is:

\begin{equation*}
C_c(jw)=C_{vw} \frac{1}{1+jw R_d C_{vw}}. \tag{25}
\end{equation*}

Regarding the proposed $\mathrm{FWK2}$, as shown in the Suppl. Materials (S.II), equation (S2), the equivalent capacitance $C_F$ of a FOC in the unit of $\mathrm{[Farad]}$, is by its very nature complex and frequency-dependent, that is:

\begin{equation*}
C_F=C_\alpha \omega ^{\beta }\left[\cos (\beta \frac{\pi }{2})+j\sin (\beta \frac{\pi }{2})\right], \tag{26}
\end{equation*}

where $\beta \!=\!\alpha \!-\!1$. It is clear from (26) that the proposed model is characterized by dynamic compliance whose modulus decreases as the frequency increases. Moreover, it follows a frequency power-law.

**Fig. 5.**
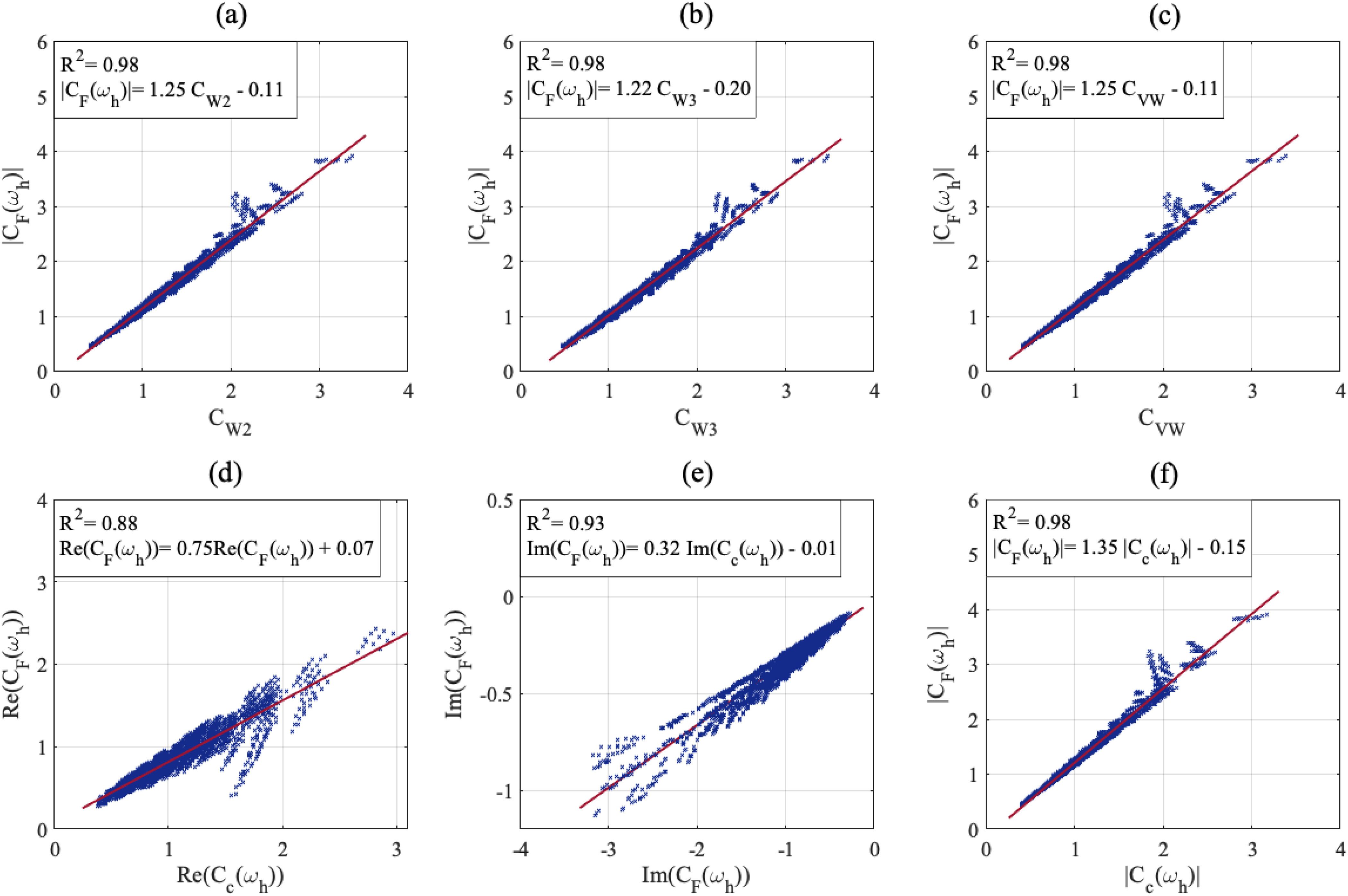
Scatter plots of the arterial compliance modulus’ estimates, $\mathrm{|C_F(\omega _h)|}$, obtained at the heart pulsation, $\mathrm{\omega _h}$, versus the corresponding estimates of: (a) $\mathrm{C_{W2}}$ the arterial compliance based WK2 model. Linear regression yielded the line: $\mathrm{|C_F(\omega _h)|=1.25 \ C_{W2}-0.11}$; $\mathrm{R^2=0.98}$, (b) $\mathrm{C_{W3}}$ the arterial compliance based WK3 model. Linear regression yielded the line: $\mathrm{|C_F(\omega _h)|=1.22 \ C_{W3}-0.20}$; $\mathrm{R^2=0.98}$, and (c) $\mathrm{C_{VW}}$ the static arterial compliance based VWK model. Linear regression yielded the line: $\mathrm{|C_F(\omega _h)|=1.25 \ C_{VW}-0.11}$; $\mathrm{R^2=0.98}$. (d) and (e) represent the correlation between the real and imaginary parts respectively of $\mathrm{C_F(\omega _h)}$ versus $\mathrm{C_c(\omega _h)}$, Linear regression yielded the line: $\mathrm{Re(C_F(\omega _h))=0.75 \ Re(C_{c}(\omega _h))+0.07}$; $\mathrm{R^2=0.88}$ and $\mathrm{Im(C_F(\omega _h))=0.32 \ Im(C_{c}(\omega _h))+0.01}$; $\mathrm{R^2=0.93}$. (f) displays the scattergram of the modulus of $\mathrm{C_F(\omega _h)}$ versus the corresponding modulus of $\mathrm{C_c(\omega _h)}$, Linear regression yielded the line: $\mathrm{|C_F(\omega _h)|=1.35 \ |C_{c} (\omega _h)|-0.15}$; $\mathrm{R^2=0.98}$.

In what follows, $|C_F(\omega _h)|$ and $|C_c(\omega _h)|$ refer to the moduli of dynamic arterial compliance determined by the $\mathrm{FWK2}$ and $\mathrm{VWK}$, respectively, at the heart pulsation, $\omega _h=2\pi / T$, where $T$ is the heart period. The scattergrams in Fig. 5 shows that $|C_F(\omega _h)|$ is correlated with the $C_{W2}$ and $C_{W3}$ which are the total arterial compliance estimates of the $\mathrm{WK2}$ and $\mathrm{WK3}$, respectively. This result indicates that the dynamic compliance evaluated at the heart pulsation by the proposed $\mathrm{FWK2}$ is consistent with the compliance of the purely elastic Windkessel. Besides, the almost perfect correlations depicted in scattergrams of Fig. 5(c) and (f), between $|C_F(\omega _h)|$ and the static compliance, $C_{VW}$ and the dynamic one $|C_c(\omega _h)|$ of $\mathrm{VWK}$, demonstrates the agreement between the viscoelastic concept and the fractional-order paradigm framework. In addition, we evaluated in Fig. 5(d) and (e) the correlation of the real and imaginary parts of the dynamic compliance-based $\mathrm{FWK2}$ at the heart pulsations versus the corresponding parts based $\mathrm{VWK}$ model. Overall, the above results prove that the $\mathrm{FWK2}$ model is appropriate in representing the dynamic compliance and is consistent with what is commonly understood in compliance physiology.

### B. Next Steps for the Fractional Windkessel Modeling

The simple model representation proposed here should be developed a little further before its generalization in the hemodynamic modeling context. First, the use of real data would considerably give more credibility to the new paradigm. In addition, the presented approach should be conducted in a range of different real physiological situations and show a good fitting for all the cases. Second, although the goal of this study is the investigation of fractional-order tools to model the aortic input impedance in the frequency domain, it is crucial to show the prediction of the pressure waveform from flow using the proposed model. Finally, It is straightforward to use FOC in the simple model representation proposed here, but there is no explicit agreement on the exact physiological relevance of the new parameter, the fractional differentiation order $\alpha$. Although it is evident from mathematical equations that $\alpha$ value controls the viscosity as well as the elasticity levels, it would be of great potential for clinical application, to define ranges of the $\alpha$ value for normal and pathological physiological conditions. As limited data are available and *in-vivo* experiments are not practical with regards to human viscoelasticity assessment, it is relevant to study the correlations between $\alpha$ changes and parameters related to the arterial stiffness effects and dependencies. Moreover, the further steps in fractional-order based aortic input impedance and hemodynamic studies should involve a more detailed distributed analog structure that could predict and explain the fractional-orders behavior based on vessel morphology and fluid blood dynamics. Subsequently, such a fractional-order arterial model would be valuable to study the effects of specific cardiovascular pathologies upon changes in the dynamic arterial compliance represented by the FOC. Besides, it might be applied to characterize aortic valve dynamics and blood flow during systole [32].

## V. Conclusions

It is commonly recognized that assessing the vascular impedance at the level of the ascending aorta provides valuable information about the physiological state of the cardiovascular system. In this study, we investigated the arterial Windkessel model within a fractional-order modeling framework. Tools from fractional-order calculus, such as the fractional-order impedance, have been used to estimate the aortic input impedance. The study focused on the $\mathrm{FWK2}$, which includes a fractional-order capacitor. The latter represents both the resistance and compliance properties, reflecting the viscoelastic behavior of the systemic arteries. The contribution of both characteristics can be controlled by the fractional differentiation order, $\alpha$, enabling a reduced-order and flexible representation of the arterial impedance. The validation and comparison with the conventional $\mathrm{WK}$ models results show how the proposed model fits better the moduli of the aortic input impedance and fairly approximates the phase angle. Besides, the proposed model's modulus dynamic compliance at the heart pulsation demonstrated a good correlation with both the static and dynamic compliance of the classical Windkessel models.

In the future, we aim to demonstrate how fractional-order arterial lumped parametric models can easily include the complex effects and the multi-scale features of arterial tissue. This new paradigm deserves consideration as a new framework for solving hemodynamics problems, and in particular, for better understanding of the arterial stiffness dependencies.

## Supplementary Materials

The supplementary information is described in more detail in the Supplementary Materials file, which includes the following sections: (S.I) Arterial Windkessel models section provides an overview of the ordinary arterial Windkessel models used for comparison in this study, (S.II) Fractional-order capacitor section provides a detailed description of FOC element, and Appendixes.


