# Supplementary material for: Assessment of Fractional-Order Arterial Windkessel as a Model of Aortic Input Impedance
Source: IEEE Open J Eng Med Biol. 2020 Apr 22;1:123–32. doi: 10.1109/OJEMB.2020.2988179 (PMC8974663; doi:10.1109/OJEMB.2020.2988179)
Supplement: Supplementary file 1 [file supp1-2988179.pdf]

Supplementary Materials

Assessment of Fractional-Order Arterial Windkessel as a Model of Aortic Input Impedance

Mohamed A. Bahloul, *Member, IEEE,* and Taous-Meriem Laleg-Kirati, *Associate Member, IEEE*

S.I. Arterial Windkessel models

Initially formulated by Frank in 1899 [*S1*], the first concept of WK, known as two-element Windkessel (WK2), considers the whole arterial network as an elastic reservoir that receives as input, a pulsatile blood flow, from the heart, and transforms this blood flow into a steady-state flow midst the resistance vessels (arterioles). As depicted in Fig. S1 (A), the electrical analog of WK2 consists of a capacitor (C_{W2}) connected in parallel to a resistor (R_p) representing, respectively, the total arterial compliance and the peripheral resistance. Although WK2 is the simplest WK configuration, with two physiolog- ically interpretable parameters, this model fails to reproduce the natural spectrum of the aortic input impedance in the frequency domain. Additionally, the estimated blood pressure is unlike the real pulse over the cardiac period. To overcome these limitations, an improved WK model has been proposed, referred to as three-element Windkessel (WK3). As shown in Fig. S1 (B), an additional series resistance (Z_c) has been connected in the inlet of WK2 to construct WK3. Despite its improvement in both frequency and time domains, WK3 repre- sents a fundamental issue that deserved additional attention in subsequent works. Indeed, the additional element, (Z_c), does not have a unique explicit physiological interpretation. The three most relevant interpretations that were attributed to it are:
1) the characteristic impedance, 2) the aortic valve resistance, and 3) the internal resistance of the left ventricle. Conceptually, these three different determinations lead to different paradigms which do not necessarily reproduce the global behavior of the arterial after-load. For instance, based on the second and third interpretations, it is obvious that Z_c belongs to the source part (heart) and hence, should not be a part of the arterial tree described by WK [*S2*]. To avoid this discrepancy and by the fact that arteries are more likely viscoelastic rather than pure elastic, a modified Windkessel model has been proposed, referred to as viscoelastic Windkessel (VWK). As depicted in Fig. S1 (C), VWK consists of a complex and frequency- dependent compliance (C_c) parallel with the total peripheral resistance (R_p). C_c is based on the electrical analogue of the Voigt mechanical cell [*S3*, *S4*]. It consists of a small resistance (R_d) and an ideal capacitor (C_{vw}) connected in series and accounting for the viscous losses of the arterial wall motion and the static compliance, respectively. It is worth to note that, mathematically, WK3, and VWK are equivalent in terms of data fitting performance; however, physiologically, they lead to different interpretations. Even though, many studies have argued that the Voigt representation is a very poor configuration of the vascular viscoelasticity, since it does not account for the stress-relaxation experiment, yet, this concept is commonly recognized as an acceptable global description of the dynamic of the arterial tree. This is related to the fact that, even if

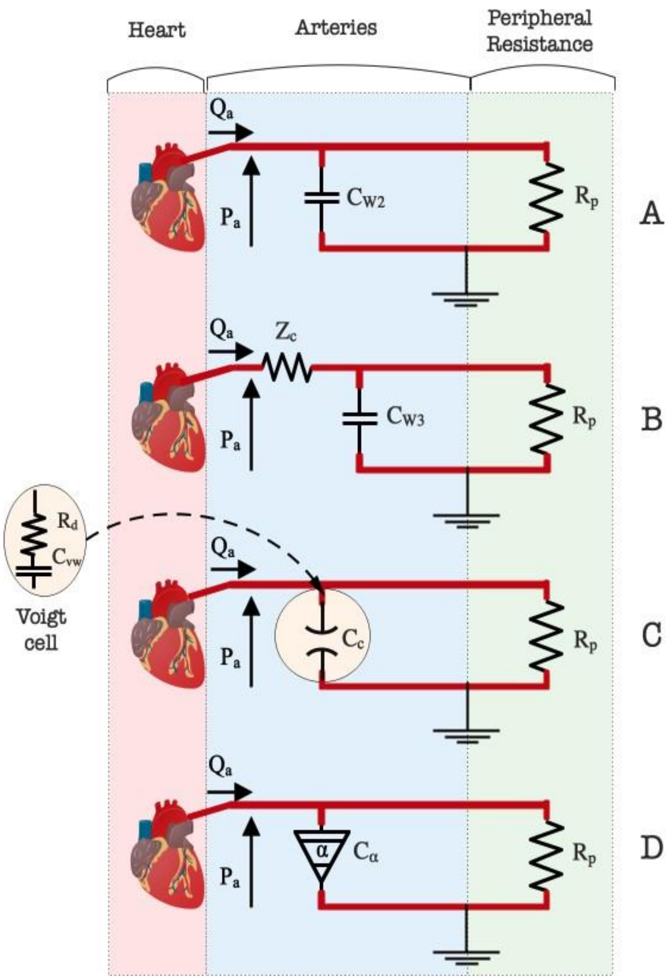

Fig. S1: Schematic representation of the electrical analog of the ordinary arterial Windkessel models (A, B, and C) along with the proposed fractional-order arterial Windkessel model (D) (adopted from Fig. 1 in [*S4*]). (A) Two-element Windkessel, (B) Three-element Windkessel, (C) Viscoelastic Windkessel, and (D) Fractional-order two-element Windkessel models. C_{W2} and C_{W3} account for the total arterial compliance in WK2 and WK3, respectively. C_C represents the frequency-dependent compliance that consists of small resistance R_d in series with an ideal capacitor C_{VW} accounting for the viscous losses and static compliance of the arterial wall, respectively. R_p represents the total peripheral resistance, and Z_c accounts for the characteristic impedance. Q_a is the arterial blood flow pumped from the left-ventricle of the heart, and P_a refers to the aortic blood pressure.

higher-order viscoelastic models would provide a more natural and realistic representation, real data cannot depict sufficient information to identify all their parameters [*S3*].

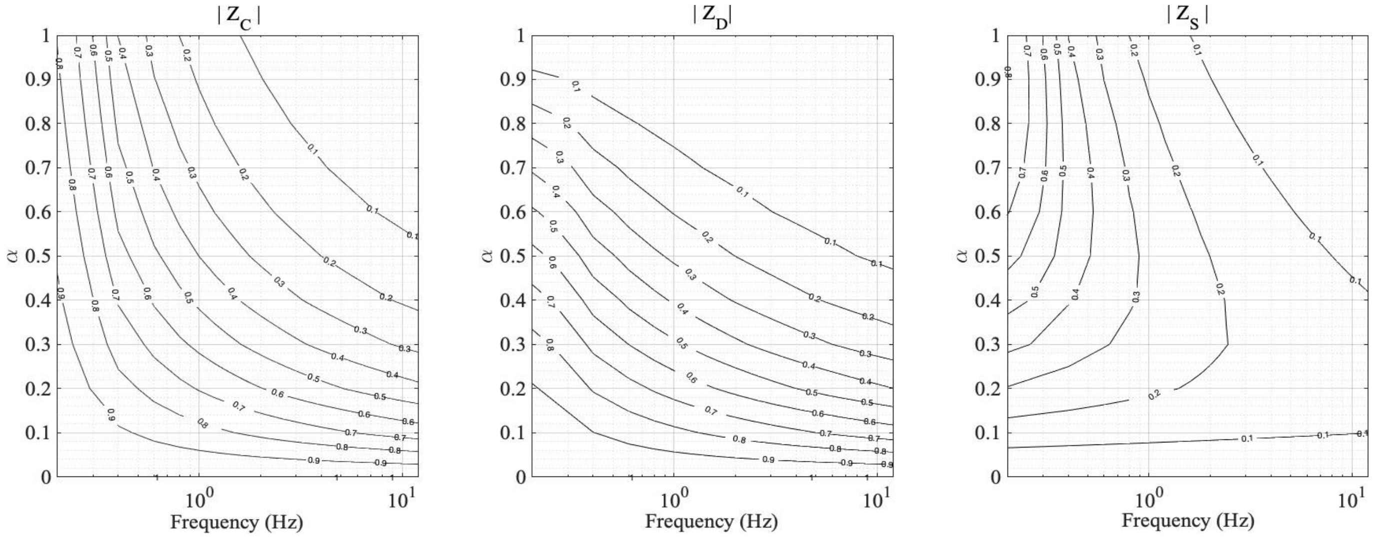

Fig. S2: Modulus of (FOC impedance Z_C, left side), (the dissipation part Z_D) and (the storage part Z_S,right side) for $C_\alpha = 1$.

S.II. Fractional-order capacitor

The fractional-order capacitor (FOC), defined as a constant phase element [*S5*, *S6*], is an electrical component that represents a fractional-order derivative relationship between the current (i), passing through, and the voltage (v), across it, with respect to time (t), that is:

$$i(t) = C_\alpha D_t^\alpha v(t), \quad \text{(S1)}$$

where C_α is a constant called pseudo-capacitance, expressed in units of [Farad ⁄ second$^{1-\alpha}$]. The capacitance in unit of [Farad] of a FOC, at a particular frequency ω_0, can be expressed as:

$$C = C_\alpha \omega_0^{\alpha-1}. \quad \text{(S2)}$$

Assuming a null initial condition and applying Laplace transform to (S1), the fractional-order impedance (Z_C) of FOC can be written as:

$$Z_C^{FOC}(s) = \frac{1}{C_\alpha s^\alpha}. \quad \text{(S3)}$$

Substituting s by ($j\omega$), (S3) becomes:

$$Z_C^{FOC}(w) = \underbrace{\frac{1}{C_\alpha \omega^\alpha}\cos(\varphi)}_{Z_D} - j\underbrace{\frac{1}{C_\alpha \omega^\alpha}\sin(\varphi)}_{Z_S}, \quad \text{(S4)}$$

where φ represents the phase shift given by the formula: $\varphi = \alpha\pi/2$ [rad] or $\varphi = 90\alpha$ [degree or $^\circ$]. As illustrated in Fig. S2, it is clear that as α goes to 0, the imaginary part (Z_S) of Z_C vanishes to 0 and hence the FOC characteristic becomes more like that a pure resistor, whereas as α approaches to 1, the real part (Z_D) converges to 0 and hence, FOC operates as a pure capacitor. Furthermore, it has been demonstrated that the characteristics of FOC can be approximated using the RC ladder structure [*S7*] similar to the one shown in Fig. S3.

with the aortic input impedance modeling concept, FOC can be considered as a suitable candidate for the complex and frequency-dependent compliance, which might overcome the discrepancies stemming from integer-order limitation. In fact,

- The proportionality constant C_α (pseudo-capacitance) is expressed in unit of [Farad.sec$^{1-\alpha}$] that makes, by its very nature, the equivalent capacitance, in the unit of [Farad], frequency-dependent as shown in (4), hence FOC provides physical foundation in representing the complex and frequency dependence of the arterial compliance.
- Based on the fractional differentiation order α, the storage and the dissipation parts of the resultant FOC's impedance can have different levels as illustrated in fig. S2, thus FOC might offer a key advantage in modeling complex systems, that is the whole spectrum of dissipation and storage mechanisms may be included in a single parameter (the fractional differentiation order).
- As depicted in fig. S3, the equivalent analogue circuit of FOC can be viewed as an infinite number of voigt cells connected in parallel, hence FOC might lead to a reduced order representation of the mechanical properties of the arterial network by using only two parameters (α and C_α).

Based on the above properties and in comparison to an integer order model where α is strictly fixed to an integer (0 or 1), the parameter α offers extra flexibility for a fractional order lumped-element modeling. In connection

APPENDIX A
FRACTIONAL-ORDER DERIVATIVE

The concept of FD is not new dating from the illustrious conversation between *L'Hopital* and *Leibniz* in 1695. FD is

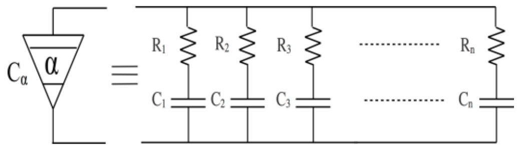

Fig. S3: RC network for fractional order capacitor emulation.

defined as a generalization of the integer order differentiation to a non-integer, real order [S8], that is:

$$D_t^{\alpha} = \begin{cases} \frac{d^{\alpha}}{dt^{\alpha}} & if \quad \alpha > 0 \\ 1 & if \quad \alpha = 0 \\ \int_0^t (d\tau)^{-\alpha} & if \quad \alpha < 0 \end{cases} \qquad \text{(A1)}$$

Where D_t^{α} is the fractional-order (differ-integration) operator, ($\alpha \in \mathbb{R}$) is an arbitrary order of the operator (integral or derivative) known as the fractional differentiation order or fractional power, and *df* is the derivative function. Several FD based mathematical formulations have been presented in the literature. Generally, these definitions can be classified into two main classes. In the first class, the operator D_t^{α} is converted into the ordinary differential-integral operator when α is integer. For instance, the Reimann-Liouville definition of a fractional-order derivative α of a function $g(t)$ is given by [S9]:

$$D_t^{\alpha} g(t) = \frac{1}{\Gamma(1-\alpha)} \frac{d}{dt} \int_0^t \frac{g(\tau)}{(1-\tau)^{\alpha}} d\tau, \qquad \text{(A2)}$$

where Γ is the Euler gamma function. The second class is that the Laplace transform of D_t^{α} is s^{α}, assuming null initial fractional conditions. The fractional operator is given by:

$$D_t^{\alpha} g(t) \xrightarrow{L} s^{\alpha} G(s), \qquad \text{(A3)}$$

This class is interesting in developing parametric models for complex systems in frequency domain. The Fourier transform can be found by substituting the complex Laplace variable (s) by ($j\omega$) and thus the equivalent frequency-domain expression of s^{α} is:

$$(j\omega)^{\alpha} = \omega^{\alpha} \left(\cos \frac{\alpha\pi}{2} + j \sin \frac{\alpha\pi}{2} \right), \qquad \text{(A4)}$$

Appendix B
Arterial Windkessel Models' Identification

The notion of identifiability is a fundamental aspect of the modeling process. Generally, for biological model the concept of identifiability is very crucial, due to the fact that the parameters of interest are representative of physiological attributes. Basically, a model is identifiable if it is theoretically possible to obtain a unique solution for the unknown parameters from experimental observations performed on the real system. Functionally, to obtain an accurate estimates of the hemodynamic model's characteristic parameters, the structural identifiability condition is a necessary condition. For integer-

A. Identification of WK2 parameter estimates

The following general *one-pole* function applies to WK2 model's structure for characterization of the aortic input impedance transfer function in the ascending aorta as ratio of polynomials in the power of s (Laplace operator):

$$H_{1p}(s) = \frac{G}{1 + \tau s}, \qquad \text{(B1)}$$

This polynomial is characterized by two observational coefficients (G, τ) that are can be determined from the experimental data, uniquely. Besides, the transfer function that characterizes the blood pressure-flow relationship-based WK2 model structure as depicted in Fig. S1 (A) can be written as:

$$Z_{in}^{WK2}(s) = \frac{R_p}{1 + R_p C_{W2} s}, \qquad \text{(B2)}$$

The relation between the observational coefficients of (B1) and the physical parameters of WK2 (C_{W2}, R_p) are given as:

$$G = R_p \qquad \text{(B3)}$$

$$\tau = C_{W2} \cdot R_p \qquad \text{(B4)}$$

Given that $G = R_p$ is evaluated as the ratio of the mean value the blood pressure to the mean value of the blood flow, the following unique solution exists for the physical parameters C:

$$C_{W2} = \frac{\tau}{G} \qquad \text{(B5)}$$

B. Identification of WK3 & VWK parameter estimates

The following general *one-pole-one-zero* function applies to WK3 and VWK models' structure for characterization of the aortic input impedance transfer function in the ascending aorta as ratio of polynomials in the power of s (Laplace operator):

$$H_{1p-1z}(s) = G \frac{1 + \tau_N s}{1 + \tau_D s}. \qquad \text{(B6)}$$

This ratio of polynomials in the power of s is characterized by three observational coefficients, denoted as G, τ_N, and τ_D which can be determined from the experimental data.

1) WK3 physical parameters' extraction: The transfer function that characterizes the blood pressure-flow relationship-based WK2 model structure as depicted in Fig. S1 (B) can be written as:

$$Z_{in}^{WK3}(s) = Z_c + \frac{R_p}{1 + R_p \cdot C_{W3} s}, \qquad \text{(B7)}$$

The following relations hold among observational coefficients (G, τ_N, τ_D) and the physical WK3 model's parameters order model, the structural identifiability analysis was studied by Bellman et. all [*S10*]. Similar analysis has been employed here to address the uniqueness of solutions for the parameters of the structure models WK2, WK3 and VWK [*S11*].

(Z_c, C, R_p):

$$G = Z_c + R_p \tag{B8}$$

$$\tau_N = \frac{Z_c \cdot R_p}{Z_c + R_p} \cdot C_{W3} \tag{B9}$$

$$\tau_D = R_p \cdot C_{W3} \tag{B10}$$

The following unique solution exists for the physical parameters (Z_c, C, R_p), that are:

$$Z_c = \frac{\tau_N \cdot G}{\tau_D} \quad \text{(B11)}$$

$$C = \frac{\tau_D}{G\left(1 - \frac{\tau_N}{\tau_D}\right)} \quad \text{(B12)}$$

$$R_p = G\left(1 - \frac{\tau_N}{\tau_D}\right) \quad \text{(B13)}$$

2) VWK physical parameters' extraction: The transfer function that characterizes the blood pressure-flow relationship-based WK2 model structure as depicted in Fig. S1 (B) can be written as:

$$Z_{in}^{VWK}(s) = R_p \frac{1 + R_d \cdot C_{VW} \cdot s}{1 + (R_p + R_d) \cdot C_{VW}}. \quad \text{(B14)}$$

The following relations hold among observational coefficients (G, τ_N, τ_D) and the physical WK3 model parameters (Z_c, C, R_p):

$$G = R_p \quad \text{(B15)}$$

$$\tau_N = R_d \cdot C_{vw} \quad \text{(B16)}$$

$$\tau_D = (R_d + R_p) \cdot C_{vw} \quad \text{(B17)}$$

The following unique solution exists for the physical parameters (R_d, C_{vw}, R_p), that are:

$$R_d = \frac{\tau_N \cdot G}{\tau_D - \tau_N} \quad \text{(B18)}$$

$$C_{vw} = \frac{\tau_D - \tau_N}{G} \quad \text{(B19)}$$

$$R_p = G \quad \text{(B20)}$$

References

[S1] N. Westerhof, N. Stergiopulos, M. I. Noble, and B. E. Westerhof, "Arterial input impedance," in *Snapshots of Hemodynamics*. Springer, 2019, pp. 195–206.

[S2] R. Burattini, "Identification and physiological interpretation of aortic impedance in modelling," in *Modeling Methodology for Physiology and Medicine*. Elsevier, 2001, pp. 213–252.

[S3] R. Burattini and S. Natalucci, "Complex and frequency-dependent compliance of viscoelastic windkessel resolves contradictions in elastic windkessels," *Medical*

[S5] M. Nakagawa and K. Sorimachi, "Basic characteristics of a fractance device," *IEICE Transactions on Fundamentals of Electronics, Communications and Computer Sciences*, vol. 75, no. 12, pp. 1814–1819, 1992.

[S6] M. A. Bahloul and T. M. Laleg-Kirati, "Three-element fractional-order viscoelastic arterial windkessel model," in *2018 40th Annual International Conference of the IEEE Engineering in Medicine and Biology Society (EMBC)*. IEEE, 2018, pp. 5261–5266.

[S7] G. Tsirimokou, "A systematic procedure for deriving rc networks of fractional-order elements emulators using matlab," *AEU-International Journal of Electronics and Communications*, vol. 78, pp. 7–14, 2017.

[S8] T. C. Doehring, A. D. Freed, E. O. Carew, and I. Vesely, "Fractional order viscoelasticity of the aortic valve cusp: an alternative to quasilinear viscoelasticity," *Journal of biomechanical engineering*, vol. 127, no. 4, pp. 700–708, 2005.

[S9] G. Jumarie, "Modified riemann-liouville derivative and fractional taylor series of nondifferentiable functions further results," *Computers & Mathematics with Applications*, vol. 51, no. 9-10, pp. 1367–1376, 2006.

[S10] R. Bellman and K. J. AAstrom, "On structural identifiability," *Mathematical biosciences*, vol. 7, no. 3-4, pp. 329–339, 1970.

[S11] K. Godfrey and J. DiStefano III, "Identifiability of model parameter," *IFAC Proceedings Volumes*, vol. 18, no. 5, pp. 89–114, 1985.

engineering & physics, vol. 20, no. 7, pp. 502–514, 1998.

[S4] Y. Aboelkassem and Z. Virag, "A hybrid windkessel-womersley model for blood flow in arteries," *Journal of theoretical biology*, vol. 462, pp. 499–513, 2019.
